# Method for obtaining the Knudsen diffusion coefficient

**DOI:** 10.1016/j.mex.2018.08.005

**Published:** 2018-08-13

**Authors:** Yoshihiko Hibi

**Affiliations:** Meijo University, Japan

**Keywords:** DGM and tracer experiment method for obtaining the Knudsen diffusion coefficient, Knudsen diffusion coefficient, Dusty gas model, Tracer experiment, Inversion simulation, Binary gas system

## Abstract

Recently, we developed a method for obtaining the Knudsen diffusion coefficient from the results of tracer experiments with a binary gas system and a porous medium in a column. The developed method employs dusty gas model (DGM) equations that consider molecular diffusion, nonequimolar fluxes, and Knudsen diffusion. The equations derived from the DGM equations for the calculation of the Knudsen diffusion coefficient can also be used to obtain the molecular diffusion coefficient and the ratio of the Knudsen diffusion coefficients of the two chemicals composing the binary gas system. We performed an inversion simulation to fit the advection–diffusion equation to the distribution of molar fractions determined by mathematically solving the advection–diffusion equation. The results confirmed that our method of obtaining the Knudsen diffusion coefficient from tracer experiment results yielded accurate values.

## Highlights

• We developed a method for obtaining the Knudsen diffusion coefficient.
• This method employs dusty gas model equations for a binary gas system.
• Equations for obtaining the Knudsen diffusion coefficient are derived.

## Graphical abstract

**Figure:**
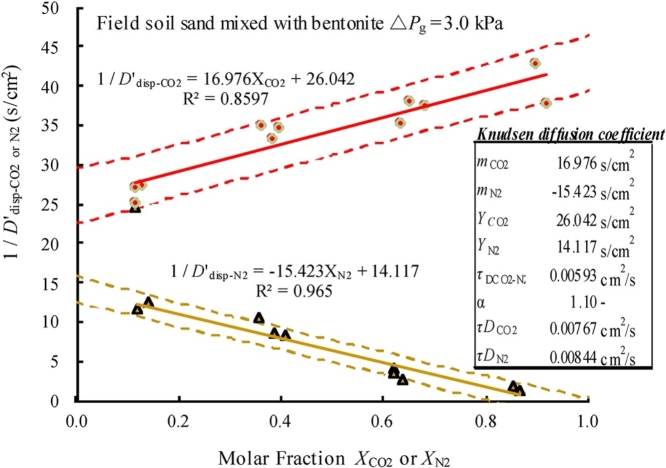

**Specifications Table**

**Table:** 

Subject area	Environmental Science
More specific subject area	Soil and Gas Pollution
Method name	DGM and tracer experiment method for obtaining the Knudsen diffusion coefficient.
Name and reference of original method	Mason, E. A., Malinauskas, A. P., Evans, R. B., 1967. Flow and diffusion of gases in porous media. Journal of Chemical Physics 46(8), 3199-3216.
	Mason, E. A., Malinauskas, A. P., 1983. Gas Transport in Porous Media: The Dusty Gas Model. Elsevier, New York, 30–50.
	Hibi, Y., Kanou, Y., Ohira, Y., 2012. Estimation of mechanical dispersion and dispersivity in a soil–gas system by column experiments and the dusty gas model. Journal of Contaminant Hydrology 131, 39-83.

## Method details

### Theoretical basis

In this section, we first present the basic theory of the DGM as it applies to a binary gas system in first section. Then we explain in second section how we derived advection–diffusion equations for calculating the effective compound diffusion coefficient and the effective compound velocity of each component gas from the DGM equations. In final section, we explain how the Knudsen diffusion coefficient of each component gas is calculated using the derived advection–diffusion equations.

#### Basic theory of dusty gas model for a binary gas system

The dusty gas model (DGM), which was developed by Mason et al. [1], can take account of the molecular diffusion flux, the Knudsen diffusion flux, and nonequimolar fluxes, and it can solve the migration of gases in multicomponent gas mixtures in a porous medium. If a gas flowing horizontally in a column filled with dry soil and a different gas is assumed, then the effect of gravity can be omitted from the equations for the migration of the first gas in the porous medium. In a binary gas system, the DGM equations are as follows:

(1a)  (*X*_A_**N***^D^*_B_ − *X*_B_**N***^D^*_A_) /*τ*_m_*θ*_g_*D*_AB_ − **N***^D^*_A_ / *τ*_p_*θ*_g_*D*_A_ = ∇*P*_A_ / *RT*

(1b)  (*X*_B_**N***^D^*_A_ − *X*_A_**N***^D^*_B_) / *τ*_m_*θ*_g_*D*_AB_ − **N***^D^*_B_ / *τ*_p_*θ*_g_*D*_B_ = ∇*P*_B_ / *RT*

where *X*_A_ and *X*_B_ are the molar fractions (dimensionless), **N***^D^*_A_ and **N***^D^*_B_ are the molar fluxes (mol/L^2^T), and *P*_A_ and *P*_B_ are the partial pressures (M/LT^2^) of component gases A and B, respectively; *R* is the gas constant (ML^2^/molKT^2^); *T* is absolute temperature (K); *D*_AB_ is the molecular diffusion coefficient between components A and B (L^2^/T); *D*_A_ and *D*_B_ are the Knudsen diffusion coefficients (L^2^/T) of components A and B, respectively; *τ*_m_ and *τ*_p_ are the tortuosity for molecular diffusion and Knudsen diffusion, respectively; and *θ*_g_ is the gas-filled porosity [[2], [3], [4]]. *τ*_m_ (0 < *τ*_m_ ≤ 1.0) is the ratio of the molecular diffusion coefficient of the porous medium to the molecular diffusion coefficient in the absence of any obstruction by solid particles. *τ*_p_ (0 < *τ*_p_ ≤ 1.0) is the ratio of the Knudsen diffusion coefficient in the DGM modified for a real porous medium to the Knudsen diffusion coefficient in the original DGM, which was developed by assuming an ideal parallel-pore arrangement and a uniform pore size [1].

#### Transformation of DGM equations to advection–dispersion equations

The molar concentrations of component gases A and B (*C_A_* and *C_B_*, respectively) (mol/L^3^) are derived from the equation of state for an ideal gas as follows:

(2a) *C*_A_ = *P*_A_ / *RT*

(2b) *C*_B_ = *P*_B_ / *RT*

The following equation is derived by summing Eqs. (1a) and (1b).

(3) **N***^D^*_A_ / *D*_A_ + **N***^D^*_B_ / *D*_B_ = − *τ*_p_*θ*_g_∇*P*_g_ / *RT*

where *P*_g_ is the total gas pressure (M/LT^2^). Eq. (3) is rearranged as follows:

(4) **N***^D^*_A_ (*D*_B_ / *D*_A_) + **N***^D^*_B_ = − *τ*_p_*θ*_g_*D*_B_∇*P*_g_ / *RT*

Then both sides of Eq. (4) are multiplied by *X*_A_ /*τ*_m_*θ*_g_*D*_AB_ to obtain Eq. (5):

(5)  (*D*_B_ / *D*_A_) (*X*_A_ /*τ*_m_*θ*_g_*D*_AB_) **N***^D^*_A_ + (*X*_A_**/***τ*_m_*θ*_g_*D*_AB_**)N***^D^*_B_ = − (*τ*_p_*D*_B_ / *τ*_m_*D*_AB_)*X*_A_ ∇*P*_g_ / *RT*

Next, Eq. (6) is derived by subtracting Eq. (5) from Eq. (1a) and substituting Eq. (2a) into the result:

(6) − (*D*_B_ / *D*_A_) (*X*_A_ /*τ*_m_*θ*_g_*D*_AB_) **N***^D^*_A_ – (*X*_B_ / *τ*_m_*θ*_g_*D*_AB_) **N***^D^*_A_ – (1 / *τ*_p_*θ*_g_*D*_A_)**N***^D^*_A_ = ∇*C*_A_ + (*τ*_p_*D*_B_ / *τ*_m_*D*_AB_)*X*_A_ ∇*P*_g_ / *RT*

Then, because *C*_A_ = *X*_A_ *P*_g_ / *RT*, Eq. (6) can be transformed as follows:

(7) − [(*D*_B_ / *D*_A_) (*X*_A_ /*τ*_m_*θ*_g_*D*_AB_) + (*X*_B_ / *τ*_m_*θ*_g_*D*_AB_**)** + (1 / *τ*_p_*θ*_g_*D*_A_)]**N***^D^*_A_*=* ∇*C*_A_ + (*τ*_P_*D*_B_ / *τ*_m_*D*_AB_)*C*_A_ ∇*P*_g_ / *P*_g_

Thus, we define the compound diffusion coefficient of component A, D^*^_A_ (L^2^/T), which includes both molecular and Knudsen diffusion, as in Eq. (7).

(8) D^*^_A_ = 1 / [(*D*_B_ / *D*_A_) (*X*_A_ / *τ*_m_*θ*_g_*D*_AB_) + (*X*_B_ / *τ*_m_*θ*_g_*D*_AB_) + (1 / *τ*_p_*θ*_g_*D*_A_)]

Therefore, by substituting Eq. (8) back into Eq. (7), and rearranging the result with respect to **N***^D^*_A_, we can obtain:

(9) **N***^D^*_A_ = − D^*^_A_∇*C*_A_ – (D^*^_A_ / *P*_g_) (*τ*_p_*D*_B_ / *τ*_m_*D*_AB_) *C*_A_∇*P*_g_

The total flux of component A, **N***^T^*_A_, is calculated from **N***^D^*_A_ (Eq. 9) and the advection flux expression –*C*_A_*k*_a_∇*P*_g_ / *μ_gmix_*, where *k*_a_ is the apparent permeability coefficient (L^2^) and *μ_gmix_* is the viscosity of the gas mixture consisting of components A and B (M/LT), as follows:

(10) **N***^T^*_A_ = − D^*^_A_∇*C*_A_– [(D^*^_A_ / *P*_g_) (*τ*_p_*D*_B_ / *τ*_m_*D*_AB_) ∇*P*_g_ + *k*_a_∇*P*_g_ / *μ_gmix_*] *C*_A_.

Here, we define V^*^_gA_ (compound velocity) (L/T) as follows:

(11) V^*^_gA_ = − [(D^*^_A_ / *P*_g_) (*τ*_p_*D*_B_ / *τ*_m_*D*_AB_) + (*k*_a_ /*μ*_gmix_)] ∇*P*_g_

We obtain Eq. (12) for **N***^T^*_A_ by combining Eqs. (10) and (11).

(12) **N***^T^*_A_ = − D^*^_A_∇*C*_A_ + V^*^_gA_*C*_A_

The mass conservation equation for component A in a porous medium is derived from **N***^T^*_A_ and *θ*_g_ as follows:

(13) *∂*(*θ*_g_*C*_A_) /∂*t* + ∇ **N***^T^*_A_ = 0

Next, Eq. (12) is substituted into Eq. (13) to obtain Eq. (14):

(14) *∂*(*θ*_g_*C*_A_) / ∂*t* +∇ ∙ (V^*^_gA_*C*_A_) = ∇ ∙ (D^*^_A_∇*C*_A_)

The first and second terms on the left side of Eq. (14) can be differentiated as follows:

(15) *θ*_g_*∂C*_A_ /∂*t* + V^*^_gA_ ∙ ∇*C*_A_ + *C*_A_ (*∂θ*_g_/∂*t* + ∇ ∙ V^*^_gA_) = ∇ ∙ (D^*^_A_∇*C*_A_).

Finally, by substituting the mass conservation equation for the total gas flux V^*^_gA_ in a porous medium, *∂θ*_g_/∂*t* + ∇ V^*^_gA_ = 0, into Eq. (15), the DGM advection–diffusion equation for component A can be derived from Eq. (15) as follows:

(16) *∂C*_A_ /∂*t* + V'^*^_gA_ ∙ ∇*C*_A_ = ∇ ∙ (*D*'^*^_A_∇*C*_A_)

where *D*'^*^_A_ and V'^*^_gA_ are the effective compound diffusion coefficient and the effective compound velocity of component A, respectively, and *D*'^*^_A_ = D^*^_A_/*θ*_g_ and V'^*^_gA_ = V^*^_gA_/*θ*_g_. By referring to Eqs. (8) and (11), These coefficients can be expressed as follows:

(17) *D*'^*^_A_ = 1 / [(*D*_B_ / *D*_A_) (*X*_A_ / *τ*_m_*D*_AB_) + (*X*_B_ / *τ*_m_*D*_AB_) + (1 / *τ*_p_*θ*_g_*D*_A_)]

(18) V'^*^_gA_ = − {[(D^*^_A_ / *P*_g_) (*τ*_p_*D*_B_ / *τ*_m_*D*_AB_) + (*k*_a_ /*μ*_gmix_)] ∇*P*_g_} / *θ*_g_.　

The advection–diffusion equation of component B is similarly derived:

(19) *∂C*_B_ /∂*t* + V'^*^_gB_ ∙ ∇*C*_B_ = ∇ ∙ (*D*'^*^_B_∇*C*_B_)

where *D*'^*^_B_ = D^*^_B_ / *θ*_g_ and V'^*^_gB_ = V^*^_gB_ /*θ*_g_ are the effective compound diffusion coefficient and the effective compound velocity of component B, respectively, which similar to those of component A can be expressed as follows:

(20) *D*'^*^_B_ = 1 / [(*D*_A_ / *D*_B_) (*X*_B_ / *τ*_m_*D*_AB_) + (*X*_A_ / *τ*_m_*D*_AB_) + (1 / *τ*_p_*D*_B_)]

(21) V'^*^_gB_ = − {[(D^*^_B_ / *P*_g_) (*τ*_p_*D*_BA_ / *τ*_m_*D*_AB_) + (*k*_a_ /*μ*_gmix_)] / *θ*_g_} ∇*P*_g_

#### Calculation of the Knudsen diffusion coefficients

To calculate the Knudsen diffusion coefficients, we first calculate the reciprocals of the effective compound diffusion coefficients (see Eqs. 17 and 20) and then substitute *X_B_* = 1 − *X_A_* and *α* = *D*_B_ / *D*_A_ into the result:

(22) 1 / *D*'^*^_A_ = (*α* − 1)*X_A_* / *τ*_m_*D*_AB_ + 1 / *τ*_m_*D*_AB_ + 1 / *τ*_p_*D*_A_

(23) 1 / *D*'^*^_B_ = (1/*α* − 1)*X*_B_ / *τ*_m_*D*_AB_ + 1 / *τ_m_D*_AB_ + 1 / *τ*_p_*D_B_*

Eqs. (22) and (23) explicitly indicate that *X*_A_ and *X*_B_ are proportional to 1 / *D*'^*^_A_ and 1 / *D*'^*^_B_, respectively. If the slope of the proportional relationship between *X*_A_ and 1/*D*'^*^_A_ is *m*_A_ and that between *X*_B_ and 1/*D*'^*^_B_ is *m*_B,_ then *m*_A_ and *m*_B_ must be as follows:

(24a) *m*_A_ = (*α* − 1) /*τ_m_ D*_AB_

(24b) *m*_B_ = (1 − *α*) /*α τ_m_ D*_AB_.

Next, Eq. (24b) is multiplied by *α* to obtain Eq. (25) as follows:

(25) *α m*_B_ = − (*α* − 1) / *τ_m_ D*_AB_.

Then Eqs. (24a) and (25) are summed to get Eq. (26):

(26) *m*_A_ +*α m*_B_ = 0

Thus, *α* is

(27) *α* = − *m*_A_ / *m*_B_.

By substituting Eq. (27) into Eq. (24a) and rearranging, *τ_m_ D*_AB_ can be obtained from the slopes of the respective proportional relationships as follows:

(28) *τ_m_ D*_AB_ = − (*m*_A_ + *m*_B_) / (*m*_A_*m*_B_).

Though Eq. (27) can be used to calculate *α*, in this method, here *α* is calculated from *m*_A_ and *τ_m_ D*_AB_ by Eq. (29), which is obtained by rearranging Eq. (24a):

(29) *α* = *m*_A_*τ_m_ D*_AB_ + 1.

The intercepts of the regression lines between *X*_A_ and 1 / *D*'^*^_A_ and between *X*_B_ and 1 / *D*'^*^_B_ are defined as

(30a) *Y*_A_ = 1/ *τ_m_ D*_AB_ + 1/*τ*_p_*D*_A_

(30b) *Y*_B_ = 1/*τ_m_ D*_AB_ + 1/*τ*_p_*D*_B_,

respectively. Eq. (30b) is then subtracted from Eq. (30a) to obtain Eq. (31):

(31) *Y*_A_ − *Y*_B_ = (1 / *D*_A_ – 1 / *D*_B_) (1 / *τ*_p_).

Next, *D*_B_ = *αD*_A_ is substituted into Eq. (31), and the result is rearranged to obtain Eq. (32):

(32) *Y*_A_ − *Y*_B_ = (1 – 1 / *α*) (1 / *τ*_p_*D*_A_).

Then Eq. (32) is rearranged:

(33) *τ*_p_*D*_A_ = (α − 1) / *α* (*Y*_A_ − *Y*_B_).

Finally, *τ*_p_*D*_B_ is derived from *α = τ*_p_*D*_B_ / *τ*_p_*D*_A_ as follows:

(34) *τ*_p_*D*_B_ = *ατ*_p_*D*_A_.

## Experimental procedure and method protocols

### Tracer experiments in a column

In Section 2.1, we describe the tracer gas experiments conducted previously [5]. Then, in Section 2.2, we explain how an inversion simulation is used to fit the advection–diffusion equation to the experimentally obtained molar fraction distribution of a tracer gas for obtaining the effective compound diffusion coefficient and the effective compound velocity of that gas. In Section 2.3, the protocol for obtaining the Knudsen diffusion coefficient of tracer gases A and B from *D*'*_A_ and *D*'*_B_ is described

**Fig. 1:**
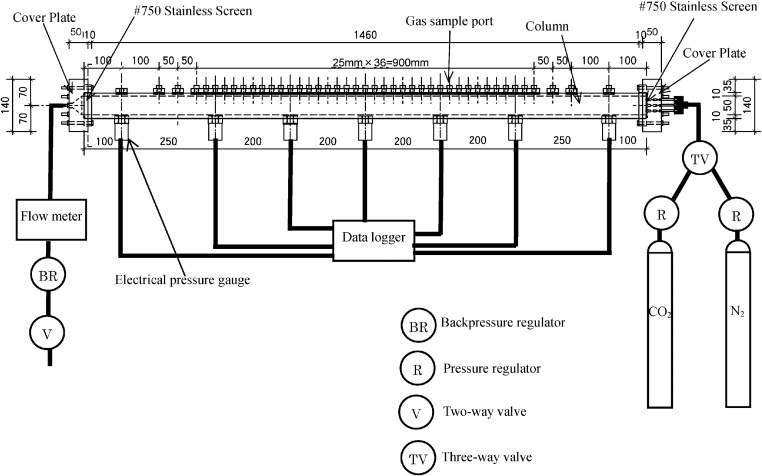
Schema of the experimental set-up with a column 150 cm long (all units of length are mm) [5].

Tracer experiments were conducted using columns of two different lengths made of acrylic resin [5]. For experiments with a rapidly diffusing tracer gas, a column with an outer diameter of 7 cm, an inner diameter of 5 cm, and a length of 150 cm was used (Fig. 1). This column was equipped with seven electrical pressure gauges (Tokyo Sokki Kenkyujo Co., Ltd., PW-100 kPa; range, 0–100 kPa; accuracy, 0.005 kPa) and 43 gas sample ports, each of which consisted of a connector (Koyo Co., Ltd., RGB 05,818) and a septum (Hamilton 9 mm DIA 12/PK) (Fig. 1). For experiments with a tracer gas with a slowly diffusion tracer gas, a column with a length of 90 cm long and the same outer and inner diameters was used. This column was equipped with five electrical pressure gauges, installed at 20-cm intervals between 5 and 85 cm from one end of the column, and 17 gas sample ports, installed at 5-cm intervals from one end of the column. The pressure gauges of each column were connected to a data logger (Tokyo Sokki Kenkyujo Co., Ltd., TDS-303), and the gas pressure in the column was measured automatically. In each experiment, gases were injected via the pressure regulators (Fairchild Model 10; range, 0–15 kPa) into the column from gas tanks (Fig. 1, right side). The gases flowed through the column (from right to left in Fig. 1) and were released to the atmosphere (Fig. 1, left side) after first passing through a flow meter (Horiba Stc Co., Ltd., SEC-N100; maximum discharge, 300 cc/min; accuracy, 0.1 cc/min) and a backpressure regulator (Fairchild Model 10BP; maximum pressure, 15 kPa). The tracer experiments were conducted at a temperature of 20 °C and a humidity of 50%. Each column was filled with a porous medium by placing it vertically, with cover plates and flow meter removed, on the laboratory floor. The filled column was then tightly closed with the cover plates and placed horizontally on a table.

**Fig. 2:**
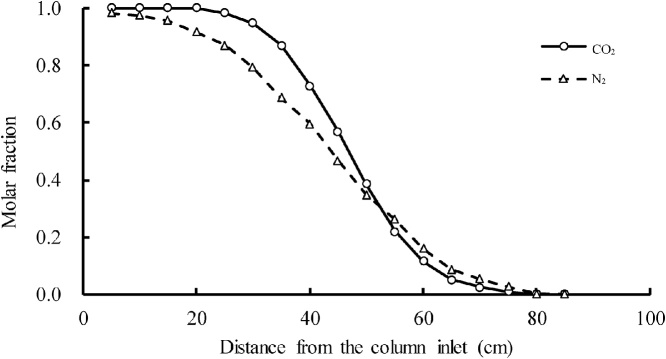
Distributions of the molar fractions of two tracer gases (CO_2_ and N_2_).

In each experiment, a gas was first injected into the column until it filled the void volume in the porous medium. It was confirmed that this gas, called the background gas, had filled the column by using gas chromatography (GL-Sciences Inc., GC-3200 with a thermal conductivity detector; carrier gas, helium; oven temperature, 50 °C; temperature in the injector, 50 °C; temperature in the detector, 70 °C) to analyze samples of gas from the column. Gas pressures at the inlet and outlet were adjusted to predetermined values by means of a gas pressure regulator installed at the inlet (Fig. 1, right side) and a gas backpressure regulator installed at the outlet (Fig. 1, left side). After it was confirmed that the column was filled with the background gas, a second gas, called the tracer gas, was injected into the column via the three-way valve. As the tracer gas was being injected into the column, gas pressure, barometric pressure, and the discharge of gas from the column were measured by the electrical pressure gauges, a barometer (Isuzu Seisakusho CO., LTD. Aneroid Barometer B-180-NO; range, 930–1070 hPa; accuracy, 1 hPa), and the flow meter, respectively. Then 0.15-mL gas samples were withdrawn from the column via the gas sample ports with syringes (SGE Analytical Science; maximum volume, 1.0 mL; minimum volume, 0.02 mL) at a specific time. The gas sampling did not affect the flow of the tracer gas in the column because only 0.15 mL of gas was sampled from each gas sample port and because the samples were collected immediately before the end of each experiment. These gas samples were analyzed by gas chromatography to determine the molar fraction of the tracer gas at each sample port at the time of sampling. Then the tracer gas and the background gas were exchanged, and another tracer experiment was conducted in the same way. For more accurate determination of the Knudsen diffusion coefficient, two to five tracer experiments were conducted with each tracer gas, one for each predetermined pressure difference between the inlet and outlet of the column. The distributions of the molar fractions of the two tracer gases were obtained from the results of these two sets of tracer experiments. For instance, the experimentally obtained distributions of the molar fractions of nitrogen and carbon dioxide (used as tracer gases) when the porous medium was oven-dried field soil (collected at Inazawa City, Japan; particle density, 2.61 g/cm^3^; porosity, 0.533; particle diameter, 0.009 to 0.7 mm) are shown in Fig. 2.

### Inversion simulation

The effective compound diffusion coefficients and the effective compound velocities of two tracer gases (gases A and B) are obtained by using an inversion simulation to fit the advection–diffusion equation to the experimentally determined distribution of the molar fraction of each tracer gas. Although for this fitting we used the ISCCFEM-adq inversion simulator developed by Hibi and colleagues [6,7], which employs a conjugate direction method [8] and a characteristic finite element scheme [9], any general inversion simulator for the advection–diffusion equation can be employed to obtain the effective compound diffusion coefficients and the effective compound velocities of the tracer gases from tracer experiment results.

The length of the one-dimensional domain for the inversion simulation, which is the length of the column used (i.e., 90 and 150 cm), is first divided into four blocks (Block 1, molar fraction 0–0.25; Block 2, molar fraction 0.25–0.50; Block 3, molar fraction 0.50–0.75; and Block 4, molar fraction 0.75–1.0). The domain grid is composed of cells, each with a length of 5 mm. The inversion simulation is then conducted for each block, and then the effective compound diffusion coefficient *D*'^*^_A_ and the effective compound velocity V'^*^_gA_ of tracer gas A in that block are obtained from the molar fraction distribution within the molar fraction range of the block (e.g., the molar fraction range of Block 1 is 0.0 to 0.25). In the simulation, there are 70 time steps from the initial distribution to the final observed distribution of concentrations. *D*'^*^_B_ and V'^*^_gB_ of tracer gas B in each block are similarly obtained with the inversion simulator from the experimentally determined molar fraction distribution of tracer gas B.

### Protocol for obtaining the Knudsen diffusion coefficients

Here we describe the protocol for obtaining the Knudsen diffusion coefficients of each tracer gas from the effective compound diffusion coefficient values determined by the inversion simulation for each molar fraction block. First, the reciprocals of the *D*'^*^_A_ and *D*'^*^_B_ values are plotted against the molar fraction of the tracer gas A and B, respectively. Then, regression lines are fitted by the method of least squares to the relationship between the molar fraction of tracer gas A, *X*_A_, and 1/ *D*'^*^_A_ and that between the molar fraction of tracer gas B, *X*_B_, and 1/ *D*'^*^_B_, and the slope and intercept of each regression line is obtained (i.e., *m*_A_ and *Y*_A_, respectively, for gas A, and *m*_B_ and *Y*_B_, respectively, for gas B). Next, *τ_m_D*_AB_ is calculated by Eq. (28) from *m*_A_ and *m*_B_, and *α* is calculated by Eq. (29) from *m*_A_ and *τ_m_D*_AB_. Finally, the Knudsen diffusion coefficient of tracer gas A, *τ*_p_*D*_A_ is calculated by Eq. (33) from *Y*_A_, *Y*_B_, and *α*, and the Knudsen diffusion coefficient of tracer gas B, *τ*_p_*D*_B_, is calculated by Eq. (34) from *τ*_p_*D*_A_ and α.

**Fig. 3:**
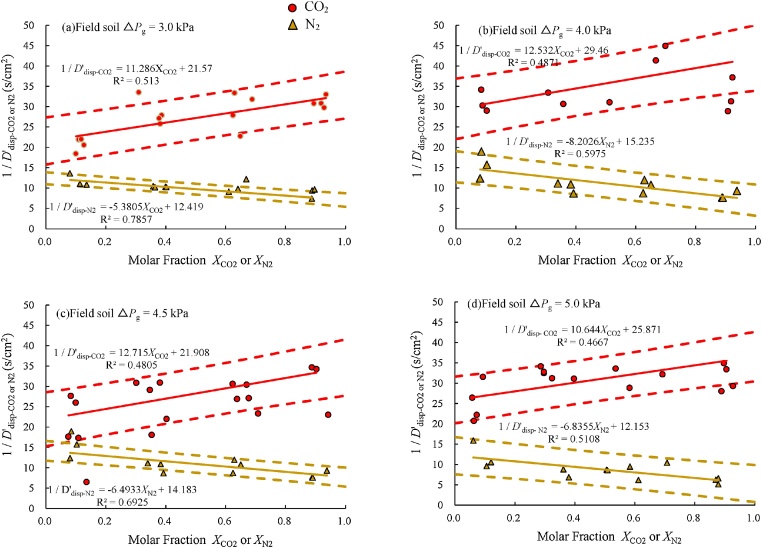
Relationships between the reciprocal of the effective compound diffusion coefficients and the molar fractions of tracer gases CO_2_ and N_2_ obtained from tracer experiments conducted with field soil (red dotted lines indicate the 95% prediction interval of CO_2_, and orange dotted lines indicate the 95% prediction interval of N_2_) [5].

For instance, in the case of the tracer experiments conducted with field soil, carbon dioxide gas, and nitrogen gas described in Section 2.1, the reciprocals of the effective compound diffusion coefficients of carbon dioxide and nitrogen, 1/*D*'^*^_CO2_ and 1/*D*'^*^_N2_, respectively, were plotted against the molar fractions of carbon dioxide and nitrogen, *X*_CO2_ and *X*_N2_, respectively (Fig. 3). When the pressure difference between the inlet and the outlet of the column was 3.0 kPa, the slope and the intercept of regression line of CO_2,_ *m*_CO2_, and *Y*_CO2_, were 11.286 s/m^2^ and 21.57 s/m^2^, respectively, and those of N_2_, *m*_N2_, and *Y*_N2_, were –5.3805 s/m^2^ and 12.419 s/m^2^, respectively (Fig. 3a). Therefore, the molecular diffusion coefficient between carbon dioxide and nitrogen, *τ_m_D*_CO2-N2_, was calculated to be 0.0973 by Eq. (28) from *m*_CO2_ and *m*_N2_, and then *α* was calculated to be 2.1 by Eq. (29) from *m*_CO2_ and *τ_m_D*_CO2-N2_. The Knudsen diffusion coefficient of CO_2_, *τ*_p_*D*_CO2_, was calculated to be 0.0572 m^2^/s by Eq. (33) from *α*, *Y*_CO2_, and *Y*_N2_. Finally, the Knudsen diffusion coefficient of N_2_, *τ*_p_*D*_N2_, was calculated to be 0.120 m^2^/s by Eq. (33) from *α* and *τ*_p_*D*_CO2_.

## Estimation of dispersion

The correlation coefficients of the regression lines, which were calculated from the coefficients of determination shown in Fig. 3, ranged from 0.68 to 0.72 for carbon dioxide and from –0.71 to –0.89 for nitrogen; thus, the reciprocal of the effective compound diffusion coefficient was correlated with the molar fraction of gas in each case. Therefore, the experimentally determined effective compound diffusion coefficient was directly proportional to the molar fraction of the tracer gas, as indicated by Eqs. (22) and (23). The effective compound diffusion coefficients obtained from the results of the tracer experiments, however, might include the effect of dispersion, which is directly proportional to the gas velocity. The effective dispersion coefficient of tracer gas A, *D***'**_disp−A_, includes the molecular diffusion coefficient *τ*_m_*D*_AB_, the Knudsen diffusion coefficient *τ*_p_*D*_A_, and the mechanical dispersion coefficient *D*_mech_. Thus, the effective dispersion coefficient *D***'**_disp−A_ of gas A can be expressed as *D***'**_disp−A_ = *D*'^*^_A_ + *D*_mech_, if the assumption of Sleep [10] that such dispersion is similar to the dispersion of chemical components dissolved in groundwater [11] is accepted. As a result, Eq. (22) becomes as follows:

(35) *D***'**_disp−A_ = 1 / [(*D*_B_ / *D*_A_) (*X_A_* / *τ_m_D*_AB_) + (*X*_B_ / *τ*_m_*D*_A_*_B_*) + (1 / *τ*_p_*D*_A_)] + *D*_mech_

The reciprocal of Eq. (35) is

(36) 1 / *D***'***_disp_*_−A_ = [(*α* − 1)*X*_A_ / *τ*_m_*D*_AB_ + 1/*τ_m_D*_AB_ + 1 / *τ*_p_*D*_A_] / {1 + *D*_mech_ [(*α* − 1)*X*_A_ / *τ*_m_*D*_AB_ + 1/*τ*_m_*D*_AB_ + 1 / *τ*_p_*D*_A_]}

**Fig. 4:**
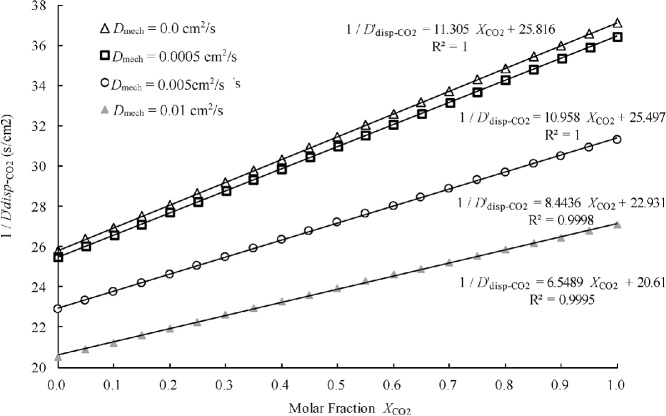
Relationships between the reciprocal of *D***'**_disp−CO2_ and *X*_CO2_ obtained by Eq. (36) with different values of *D*_mech._

*D***'**_disp−CO2_ of carbon dioxide was calculated by using Eq. (36), the values of *τ_m_D*_CO2-N2_, *α*, and *τ*_p_*D*_CO2_ obtained in Section 2.3, where gas A is CO_2_ and gas B is N_2_, and each of four different values for *D*_mech_ (0.0, 0.0005, 0.005, and 0.001 cm^2^/s). Then, 1/*D***'**_disp−CO2_ was plotted against the molar fraction of CO_2_, *X*_CO2_, as shown in Fig. 4. However, although *D***'**_disp−CO2_ appears to be directly proportional to *X*_CO2_ in the figure, the mathematical relationship between *D***'**_disp−CO2_ and *X*_CO2_ described by Eq. (36) is not rigorously proportional. In fact, the coefficient of determination of the regression lines between *D***'**_disp−CO2_ and *X*_CO2_, *R*^2^, shown in Fig. 4, decreased slightly, from 1.0000 to 0.9995, as the mechanical dispersion coefficient *D*_mech_ increased; therefore, Eq. (36) is not obviously consistent with any particular line. Furthermore, the slope of the regression line also decreased as *D*_mech_ increased, instead of remaining constant.

**Fig. 5:**
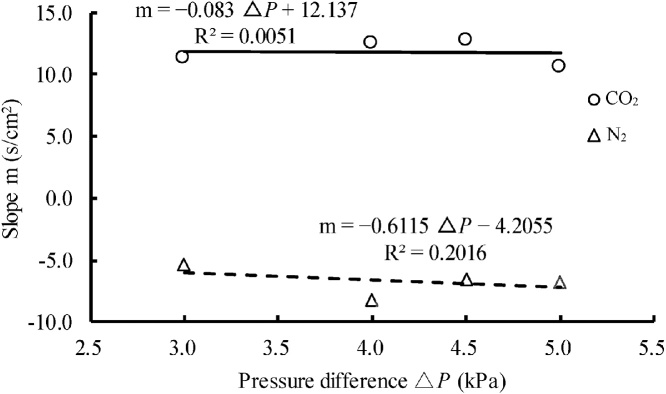
Relationships between the slope (*m*) of the regression line between 1/*D***'***_disp_*_−CO2_ and *X*_CO2_ and that between 1/*D***'***_disp_*_−N2_ and *X*_N2_, obtained by the tracer experiments, and the pressure difference Δ*P* between the inlet and outlet of the column.

In contrast, the relationships between 1/*D*'*_CO2_ and *X*_CO2_ and between 1/*D*'*_N2_ and *X*_N2_ obtained from the tracer experiment results by the protocol described in Section 2.3 could be expressed as linear equations. Further, when we plotted the slope *m* of the regression line for the relationship between 1/*D***'**_disp−CO2_ and *X*_CO2_ and the slope *m* of the regression line for the relationship between 1/*D***'**_disp-N2_ and *X*_N2_ against the pressure difference Δ*P* (Fig. 5), the *R*^2^ values of the *m*–Δ*P* relationships for CO_2_ and N_2_ were very small, 0.0051 and 0.2016, respectively. Further, the slopes of the regression lines of the *m*–Δ*P* relationships were very small, –0.083 and –0.6115, respectively; thus, *m* did not change with Δ*P* but was approximately constant and independent of Δ*P*. Because in general *D*_mech_ increases as Δ*P* increases, the slopes of the regression lines between 1/*D***'**_disp-CO2_ and *X*_CO2_ and between 1/*D***'**_disp−N2_ and *X*_N2_ were constant, regardless of the value of *D*_mech_.

Therefore, the relationship between *D***'**_disp−CO2_ and *X*_CO2_ obtained by Eq. (36) is not consistent with the trend obtained by means of the actual tracer experiment, shown in Fig. 3. On the other hand, when *τ_m_D*_AB_ in Eq. (22) is replaced with *τ_m_D*_AB_ + *D*_mech_, the slope of the regression line between *D***'**_disp−A_ and *X*_A_ can become smaller as *D*_mech_ increases. Because the slope of the regression line between 1/*D***'***_disp_*_−A_ and *X*_A_ is constant, independent of the value of *D*_mech_, mechanical dispersion must influence only the value of the intercept of Eq. (22). Consequently, the relation between *X*_A_ and 1/*D***'**_disp−A_ must be

(37) 1 / *D***'**_disp-A_ = (*α* − 1) *X*_A_ / *τ_m_ D*_AB_ + 1 / *τ*_m_*D*_AB_ + 1 / *τ*_p_*D*_A_ + Δ_mech_

Similarly, for tracer gas B, the relation between *X_B_* and the reciprocal of *D***'**_disp−_*_B_* is

(38) 1 / *D***'**_disp-B_ = (1 / *α* − 1) *X*_B_ / *τ_m_ D*_AB_ + 1 / *τ*_m_*D*_AB_ + 1 / *τ*_p_*D*_B_ + Δ_mech_

where Δ_mech_ is a parameter that we call the "difference coefficient with mechanical dispersion", the value of which depends on the properties of the porous medium. We can similarly obtain Eqs. (24) to (34) (see Section 1.3) from Eqs. (37) and (38), except for Eqs. (30a) and (30b), which become as follows:

(39a) *Y*_A_ = 1/ *τ_m_ D*_AB_ + 1/*τ*_p_*D*_A_ + Δ_mech_

(39b) *Y*_B_ = 1/*τ_m_ D*_AB_ + 1/*τ*_p_*D*_B_ + Δ_mech._

Because when Eq. (39b) is subtracted from Eq. (39a), Δ_mech_ is eliminated, Eq. (31) remains as shown in Section 1.3.

## Accuracy of the experimentally determined Knudsen diffusion coefficients

We estimated the accuracy of the method used to obtain the Knudsen diffusion coefficients from the results of tracer experiment results. Ogata and Banks [12] mathematically solved a one-dimensional advection–diffusion equation, with the boundary conditions that the concentration was *C*_0_ at *x* = 0 and *t* = 0 and 0.0 at *x* = ∞ and *t* = 0 and the initial condition that the concentration was 0.0 at *x* > 0 and *t* = 0, where *x* is the distance from the gas inlet and *t* is the elapsed time from the injection of gas as follows:

(40) *C* = (*C*_0_/2){erfc[(*x* – *Vt*) / 2(*Dt*)^1/2^] + exp(*Vx*/*D*) erfc[(*x* + *Vt*) / 2(*Dt*)^1/2^}

where *C* is the concentration of the gas, *D* is the diffusion coefficient, and *V* is the velocity of the gas. The concentration calculated with Eq. (42) should have no numerical noise, unlike solutions obtained by numerical simulation. We use the ISCCFEM-adq inversion simulator (see Section 2.2) to obtain the diffusion coefficient from the distributions of concentrations calculated by Eq. (40) with C_0_ = 1.0. The diffusion coefficient obtained by the inversion simulation should thus be equal to the diffusion coefficient *D* used in Eq. (40) for calculating the distribution of the concentrations. Therefore, the difference between the dispersion coefficient obtained by the inversion simulation and that used for calculating the distribution of concentrations is the error in the method we use to obtain the Knudsen diffusion coefficient from the tracer experiment results.

The length of the one-dimensional domain, the range used to divide the molar fraction into blocks, the cell size of the grid, and the number of time steps used in the inversion simulation were the same as those in the real tracer experiment. The length of the simulation domain was set equal to 90 cm, the length of the column used in the real tracer experiments. The inversion simulation was conducted for each block, and then the diffusion coefficient of each block was obtained from the concentrations calculated with Eq. (40). The observation points for the inversion simulations were arranged at *x* = 10, 20, and 25 cm and at intervals of 2.5 cm over the *x*  = 30 cm, consistent with the locations of the gas sampling points in the real tracer experiments.

The distribution of concentrations was calculated for three cases: a velocity of 0.05 cm/s, a diffusion coefficient of 0.1 cm^2^/s, and an elapsed time from the injection of 1200s (Case 1); a velocity of 0.02 cm/s, a diffusion coefficient of 0.02 cm^2^/s, and an elapsed time from the injection of 2000s (Case 2); and a velocity of 0.005 cm/s, a diffusion coefficient 0.01 cm^2^/s, and an elapsed time from the injection of 8000 s and 11,000 s (Case 3). Concentrations obtained by the numerical inversion simulation were then fitted to the concentrations calculated with Eq. (40) at the indicated observation points. The results show that the diffusion coefficients obtained by the inversion simulation differed from those used to compute Eq. (40) by less than 3%, with the exception of Block 4 at the elapsed time 11,000 in Case 3 (Error 8%). The *F* statistic value, which is equal to the sum of the squares of the differences between the concentrations calculated by Eq. (42) and those obtained by the inversion simulation at the observation points, ranged from 4.62 × 10^–9^ to 8.38 × 10^–5^ (Table 1). Therefore, the ISCCFEM-adq inversion simulator used in this study was able to accurately simulate the diffusion coefficient.

**Table 1:** Inversion simulation results for the distributions of concentrations calculated with Eq. (40) [5].

Input parameter for simulation (Case 1, Time 1200 s)			*V* = 0.05 cm/s *D* = 0.1 cm2/s			
	Block No.	Molar Fraction *X*	*V* (cm/s)	*D* (cm^2^/s)	1 / *D* (s/cm^2^)	*F*
Results of Inversion analysis	1	0.895	0.0497	0.0995	10.05	4.04E-07
	2	0.645	0.0498	0.1009	9.91	5.00E-09
	3	0.380	0.0498	0.1009	9.91	4.62E-09
	4	0.109	0.0498	0.0997	10.03	1.76E-07

Input parameter for simulation (Case 2, Time 2000 s)			*V* = 0.02 cm/s *D* = 0.02 cm^2^/s			
	Block No.	Molar Fraction *X*	*V* (cm/s)	*D* (cm^2^/s)	1 / *D* (s/cm^2^)	*F*
Results of Inversion analysis	1	0.899	0.0199	0.0200	50.11	2.20E-07
	2	0.606	0.0199	0.0202	49.55	1.33E-08
	3	0.394	0.0199	0.0202	49.55	1.37E-08
	4	0.100	0.0199	0.0198	50.40	1.54E-05

Input parameter for simulation (Case 3, Time 8000 s)			*V* = 0.005 cm/s *D* = 0.01 cm^2^/s			
	Block No.	Molar Fraction *X*	*V* (cm/s)	*D* (cm^2^/s)	1 / *D* (s/cm^2^)	*F*
Results of Inversion analysis	1	0.888	0.0050	0.0100	100.29	2.22E-07
	2	0.612	0.0050	0.0101	99.13	1.23E-08
	3	0.388	0.0050	0.0100	99.79	4.70E-06
	4	0.108	0.0050	0.0098	102.21	1.55E-05

Input parameter for simulation (Case 3, Time 11,000 s)			*V* = 0.005 cm/s *D* = 0.01 cm^2^/s			
	Block No.	Molar Fraction *X*	*V* (cm/s)	*D* (cm^2^/s)	1 / *D* (s/cm^2^)	*F*
Results of Inversion analysis	1	0.859	0.0051	0.0097	103.38	8.09E-07
	2	0.616	0.0051	0.0099	101.36	6.72E-08
	3	0.371	0.0051	0.0099	101.04	9.56E-09
	4	0.140	0.0051	0.0092	108.90	8.38E-05

**Fig. 6:**
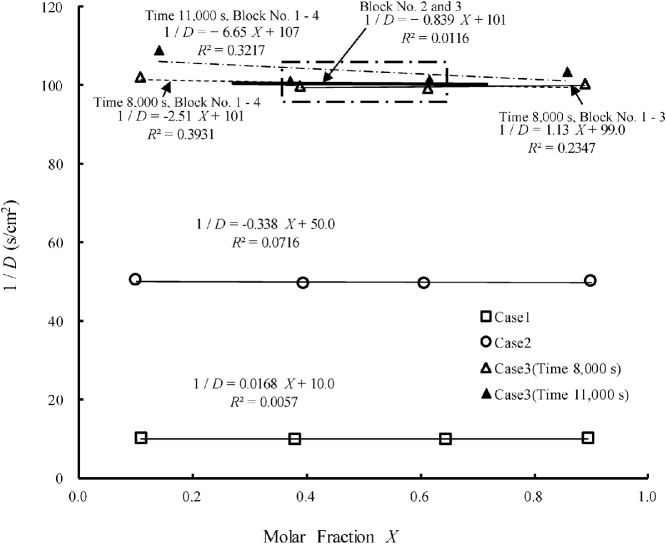
Relationships between the reciprocal of diffusion coefficient and the molar fraction obtained by inversion simulations with the concentrations calculated with Eq. (40) [5].

The slope of the regression line between the molar fraction of the tracer gas and the reciprocal of the compound dispersion coefficient is an important parameter in this method for obtaining the Knudsen diffusion coefficient. In Eq. (40), the diffusion coefficient *D* is constant, independent of the molar fraction of the tracer gas. In Cases 1 and 2, the reciprocal of the diffusion coefficient was not correlated with the molar fraction of the gas but was constant everywhere, and the slopes of the regression lines were 0.0168 and –0.338 s/cm^2^, respectively. Though the slope of the regression line for all data at the elapsed time of 8000 s in Case 3 was 2.51 s/cm^2^, the slope of the regression line for the data of Blocks 1–3 only at the elapsed time of 8000 s in Case 3, 1.13 s/cm^2^, was less than half that for all data. At the elapsed time 11,000 in Case 3, the absolute slope of the regression line was 6.65 s/cm^2^ for all data and 1.13 s/cm^2^ for Blocks 1–3 only. Thus, similar to the situation at the elapsed time 8000 s in Case 3, at the elapsed time of 11,000 s in Case 3 the absolute slope of the regression line for all data was greater than that for data of Blocks 1–3 only. Furthermore, the absolute slope of the regression for data of Blocks 2–3 at elapsed times of both 8000 s and 11,000 s in Case 3 was 0.839, the smallest slope for Case 3 data (Fig. 6). Accordingly, we confirmed that this method can accurately reproduce the slope of the regression line between the reciprocal of the effective compound dispersion coefficient and the molar fraction of tracer gas when the reciprocal of the effective compound dispersion coefficient is less than 50 s/cm^2^. However, when the reciprocal of the effective compound dispersion coefficient is more than 100 s/cm^2^, the data in Block 1 (molar fraction 0.0–0.25) and Block 4 (molar fraction 0.75–1.0) must be excluded to accurately obtain the slope of the regression line. Further, many reciprocals of the compound dispersion coefficient must be obtained by fitting the advection–diffusion equation to the data at several different elapsed times with the inversion simulator to more accurately obtain the slope of the regression line.

## Summary

To apply this method, the distributions of the molar fractions should first be obtained by conducting tracer experiments with a porous medium and a binary gas system consisting of components A and B. The tracer experiments are first conducted with component A as the tracer gas and component B as the background gas, and subsequently with component B as the tracer gas and component A as the background gas. Next, the advection–dispersion equation is fitted to the distribution of the molar fractions of tracer gas A or B obtained by the tracer experiments by means of an inversion simulation in order to obtain the effective compound dispersion coefficient and the effective compound velocity. The inversion simulation is conducted separately for four blocks (Block 1, molar fraction 0–0.25; Block 2, molar fraction 0.25–0.50; Block 3, molar fraction 0.50–0.75; and Block 4, molar fraction 0.75–1.0); thus, the effective compound dispersion coefficient and the effective compound velocity are obtained for each block. Further, the average molar fraction of each block is obtained by averaging the maximum and minimum molar fractions in the block. Then, the effective compound dispersion coefficients are plotted against the averages of the molar fractions, and two regression lines between the averages of the molar fraction and the reciprocal of the effective compound dispersion coefficient are calculated, one for tracer gas A and the other for tracer gas B, by the least-squares method. *τ_m_ D*_AB_ is calculated from the slopes of the regression lines for tracer gas A and B, *m*_A_ and *m*_B_, respectively, by using Eq. (28), and then *α* is calculated from the slope of the regression line for component A *m*_A_ and *τ_m_ D*_AB_ by using Eq. (29). *τ*_p_*D*_A_ can then be determined from *α* and the intercepts of the two regression lines between the average of the molar fractions and the reciprocal of the effective compound dispersion for tracer gas A and tracer gas B, *Y*_A_ and *Y*_B_, respectively, by using Eq. (33). Finally, *τ*_p_*D*_B_ is calculated from *α* and *τ_m_ D*_AB_ by using Eq. (34).

## Additional information

The Knudsen diffusion coefficient can be obtained from the results of permeability experiments. Reinecke and Sleep [13] and Abu-El-Sha’r and Abriola [14] actually performed permeability experiments with the aim of obtaining the Knudsen diffusion coefficient.

In the case of a compression fluid in a porous medium, apparent gas permeability *k_a_* (L^2^) in the porous medium can be obtained as follows:

(41) *k_a_* = *q* / [(*P*_1_^2^ – *P*_2_^2^) / 2*Lμ_g_P*_2_]

where *P*_1_ and *P*_2_ are the gas pressures at the inlet and the outlet, respectively, of a column filled with gas and a porous medium; *q* is the volumetric flux (L/T) discharged from the outlet; *L* is the length of the column packed with porous medium (L); and *μ_g_* is viscosity (M/LT).

Klinkenberg [15] derived the following equation, which relates effective gas permeability *k_e_* (L^2^) to apparent gas permeability *k_a_* by considering the slip effect, that gas velocity at the surface of a solid is not zero:

(42) *k_a_* = *k_e_* + *bk_e_* / *P*_g_*_ave_*

where *b* is the Klinkenberg parameter (M/LT^2^) and *P*_g_*_ave_* is the average gas pressure.

It is clear from Eq. (42) that a plot of *k_a_* against 1/ *P*_g_*_ave_* yields a straight line with slope *bk_e_* and intercept *k_e_*. The Klinkenberg parameter can be obtained by dividing the slope by *k_e_*. If chemical component A composes a single-gas system in a porous medium, then the average gas pressure can be related to the total molar flux of component A, *N^T^*_A_ (mol/L^2^ T), by employing the DGM equation for a single-component gas system as follows:

(43) *N^T^*_A_*RT* / ∇*P*_g_ = *D*_A_ + *k_e_ P*_g_*_ave_* / *μ_g_*

If the gas in the porous medium is assumed to obey Darcy’s law and the ideal gas law, the total molar flux of component A can be expressed as follows:

(44) *N^T^*_A_ = – (*P*_g_*_ave_* / *RT*)(*k_a_* /*μ_g_*)∇*P*_g_.

By rearranging Eq. (44), *N^T^*_A_*RT* / ∇*P*_g_ can be calculated with Eq. (45) as follows:

(45) *N^T^*_A_*RT* / ∇*P*_g_ = – *P*_g_*_ave_* (*k_a_* /*μ_g_*)

A plot of *N^T^*_A_*RT* / ∇*P*_g_ against *P*_g_*_ave_* in Eq. (43) yields a straight line with slope *k_e_* / *μ_g_*, and the Knudsen diffusion coefficient of component A is the intercept. The effective gas permeability can be calculated by multiplying this slope by the viscosity.
